# The morphology of the immature stages of *Metadonus vuillefroyanus* (Capiomont, 1868) (Coleoptera, Curculionidae, Hyperini) and notes on its biology

**DOI:** 10.3897/zookeys.589.7847

**Published:** 2016-05-16

**Authors:** Jiří Skuhrovec, Petr Bogusch

**Affiliations:** 1 Group Function of Invertebrate and Plant Biodiversity in Agrosystems, Crop Research Institute, Prague 6–Ruzyně, Czech Republic; 2 Department of Biology, Faculty of Science, University of Hradec Králové, Rokitanského 62, CZ-500 03 Hradec Králové, Czech Republic

**Keywords:** Weevil, mature larva, pupa, larval development, life cycle, host plant, Suaeda vera, Amaranthaceae, Spain, Palaearctic region

## Abstract

Last instar larva and pupa of *Metadonus vuillefroyanus* (Capiomont, 1868) (Curculionidae: Hyperini) are described and compared with known larvae of the other 43 hyperine taxa. The thorn-like setae located on distinct black protuberances on the larval body are characteristic features of the genus *Metadonus* and the subgenus *Eririnomorphus* of the genus *Hypera*. The biological singularity of this species was studied and described. The variable colouration of larvae has been confirmed in association with the variability of the host plant’s colouration at the studied localities. This species’ reported inability to spin cocoons has been disproven. A different type of cocoon with two layers, where the inner layer consists of proteins from Malpighian tubules while the outer layer contains soil particles, is described. This type of cocoon is unique compared with those known from other hyperines, which usually pupate on or above the ground and do not use substrate particles in building their cocoons.

## Introduction

The phylogeny and taxonomy of hyperines is still unresolved. Recently, hyperines together with Bagoini and Gonipterini have been considered unclassifiable tribes in Curculionidae (Oberprieler et al. 2014). However, the hyperines have been also treated as other groups; e.g. a subfamily Hyperinae (e.g., Thompson 1992; Zimmerman 1992; Alonso-Zarazaga and Lyal 1999; Anderson 2002; Marvaldi et al. 2002; Marvaldi and Lanteri 2005; Bouchard et al. 2011); within a subfamily Brachycerinae (Kuschel 1995); as a tribe of Curculioninae (Oberprieler et al. 2007; McKenna et al. 2009); as a tribe of Entiminae (Legalov 2011a, b); and finally, in a clade that also included Entiminae, Cyclominae and Gonipterini (Hunt et al. 2007; Hundsdoerfer et al. 2009; McKenna et al. 2009; Gunter et al. 2015).

Hyperines have characteristic shapes, but the genera recently included in the tribe have so far shown no distinct diagnostic or synapomorphic characters that would permit a satisfactory concept of Hyperini (Oberprieler et al. 2014) to be drawn up. Their only unique feature appears to be the peculiar meshed cocoon spun by the larvae from strands of protein secreted by the Malpighian tubules (Scherf 1964; Kenchington 1983). The latest attempt to define this group was conducted by Petri (1901), who defined them using ten features. However, of these, only the characters of the trochanters, claws and pygidium hold true, and none of these are unique to Hyperini (Oberprieler et al. 2014). Petri (1901) divided the Hyperini into two subtribes, Hyperina and Cepurina, based on the shape of the mesepimera and the length of the metanepisterna and the relative width and angle of their junction with the mesepimera. Legalov (2007, 2010, 2011) classified this tribe into five subtribes: Cepurina, Hyperina, Coniatina, Macrotarrhusina and Phaeopholina, based on several morphological characters, but such a distinction requires a more comprehensive study of the whole tribe and is equally unlikely to yield meaningful synapomorphies to identify family group taxa within the group (Oberprieler et al. 2014). Oberprieler et al. (2014) and Skuhrovec (unpublished data) recently divided this tribe into three “operating” groups with different distributions: (1) the Palaearctic region (Hyperina), (2) the circumtropical region (Cepurina), and (3) the Australian/Pacific region (Australian Hyperini and *Phaeopholus* Roelofs, 1873).

Several recent taxonomic studies deal with the Palaearctic fauna of Hyperini. Skuhrovec (2005a, 2005b, 2006a, 2006b, 2007) studied the larvae of *Donus* Jekel, 1865 and *Hypera* Germar, 1817, he clarified (2008) the complex nomenclature of the large and important genera *Brachypera* Capiomont, 1868, *Donus* and *Hypera*, and revised (2012) the genus *Metadonus* Capiomont, 1868. Alonso-Zarazaga and Lyal (2002) transferred the monotypic genus *Herpes* Bedel, 1874, previously classified in Brachycerinae or Rhythirrinini but in Thecesternini by Alonso-Zarazaga and Lyal (1999), to Hyperini. Legalov (2011a) recently resurrected a number of subgenera of *Coniatus* Germar, 1817, *Hypera* and *Macrotarrhus* Bedel, 1906 to generic status, but these taxonomic acts were published without detailed justification, and this is the main reason why Skuhrovec (2013a) and Oberprieler et al. (2014) did not accept these taxonomic changes. A detailed comparative study of hyperine immature stages is also necessary in this context because most larval and pupal characters are only known from the relatively well-studied genera *Brachypera*, *Donus*, *Hypera* and *Phelypera* Jekel, 1865 (Oberprieler et al. 2014), and the larvae of only a few other genera have been described (e.g., *Fronto* Petri, 1901, *Metadonus* and *Macrotarrhus*) (Zaslavskij 1959).

The genus *Metadonus* has been revised recently (Skuhrovec 2012) and now includes 10 species, all of which are known to be native to the Palaearctic region. They occur primarily in Asia, but exceptions include *Metadonus vuillefroyanus*, which is found in Spain, Morocco and Algeria, and *Metadonus anceps* (Boheman, 1842) and *Metadonus distinguendus* (Boheman, 1842), which occur in Ukraine, Moldavia, Romania, Turkey and Russia (Skuhrovec 2012, 2013b). Species of this genus live in extreme conditions (such as cold steppes, salinas and semi-deserts) (Skuhrovec 2012). Biological notes about host plants are known only for *Metadonus vuillefroyanus*, *Metadonus distinguendus* and *Metadonus anceps* (Skuhrovec 2008, 2012; Korotyaev et al. 2016). Velázquez de Castro et al. (2000) listed the first biological data about *Metadonus vuillefroyanus*, which occurs in salt wetlands; its host plant is *Suaeda vera* (synonym *Suaeda fruticosa*) (Amaranthaceae) (Skuhrovec 2012).

The immature stages of *Metadonus vuillefroyanus* are here described for the first time. Knowledge of the immature stages and the life history of a species are important for both taxonomic and applied use and can help protect this species more effectively. Taking into account the information gathered by the first author about the biology of immature stages and adults of *Metadonus vuillefroyanus*, the second author undertook a study trip to Spain. In the present paper we provide biological data based on Bogusch’s observations obtained during his field work in Spain, and we describe the immature stages of this species.

## Materials and methods

The material used to describe the immature stages was collected, and field observations were conducted in the following localities: **SPAIN: Almería**: Cabo de Gata National Park, Cabo de Gata, Salinas, surroundings of salt marshes (36°46'48"N, 2°13'44"W, 2 m), 29-III-2014, 1 ♂ and 15 larvae swept from *Suaeda vera*; 31-III-2014, 5 larvae swept from *Suaeda vera*; Tabernas env., river valley (37°02'57"N, 2°24'28"W, 339 m), 1-IV-2014, 2 mature larvae swept from *Suaeda vera*, all P. Bogusch and A. Astapenková leg., P. Bogusch det., revised by J. Skuhrovec, in the collections of P. Bogusch and J. Skuhrovec. Descriptions of immature stages were done on four larvae and two pupae.

Part of the larval and pupal material was preserved in Pampel fixation liquid (4 parts glacial acetic acid, 6 parts 4% formaldehyde, 15 parts 95% ethyl alcohol and 30 parts distilled water) and used for the morphological descriptions. These specimens are now deposited in the Group Function of Invertebrate and Plant Biodiversity in Agrosystems of the Crop Research Institute (Prague, Czech Republic). Plants were identified by the collectors. To prepare the slides we followed May (1994). The head of the larva was separated and cleared in a 10% potassium hydroxide (KOH) solution and then rinsed in distilled water. After clearing, the mouth parts were separated from the head capsule. The head capsule and all mouth parts were mounted on permanent microscope slides in Euparal. All other body parts were mounted on temporary microscope slides in 10% glycerine.

The observations and measurements were made using a light microscope with calibrated oculars (Olympus BX 40 and Nikon Eclipse 80i). The following measurements were taken for each larva: head width, length of the body (larvae fixed in a C-shape were measured along segments), width of the body in the widest place (metathorax or abdominal segments I–IV), and these for each pupa: length and width at the widest place. The thorax and abdomen were not sclerotised, and it is unlikely that the fixation process altered the weevils’ proportions; measurements of these parts are given for comparison purposes only.

Drawings were made with a drawing tube on a light microscope and processed using a computer program (Adobe Photoshop, Corel Photo-Paint 11, GIMP 2). The thoracic spiracle is placed on the prothorax near the boundary of the prothorax and mesothorax, as shown in the drawing (see Fig. 8), but it is of mesothoracic origin (Marvaldi et al. 2002; Marvaldi 2003). The drawings show the thoracic and abdominal spiracles (see Figs 8–10). The numbers of setae of the bilateral structures are given for one side.

We used the terms and abbreviations for the setae of the mature larva and pupa studied following Scherf (1964), May (1977, 1994) and Marvaldi (1998a, 1999).

## Results

### Metadonus vuillefroyanus

Taxon classificationAnimaliaColeopteraCurculionidae

(Capiomont, 1868)

Phytonomus vuillefroyanus Capiomont, 1868: 135

#### Description of mature larva.

*Measurements* (in mm). Body length: 10.0–14.0 (mean 12.0). The widest place in the body (abdominal segments II–VI) measures up to 2.5. Head width: 0.9–1.1 (mean 1.0).

*Colouration*. Dark brown to black head (Fig. 7). All thoracic and abdominal segments greenish with white longitudinal stripes on both sides of body, but this larva also has a thick longitudinal yellow stripe and parallel longitudinal pink to violet stripes in its dorsal part with small black short stripes inside; all setae are thorn-like, located on distinct black protuberances in very thin white transversal lines (Figs 7–10, 16).

*Vestiture*. Body elongated, slightly curved, rounded in cross section (Fig. 7). Setae on body thin, different in length (short to relatively long), black thorn-like, located on distinct black protuberances.

**Figure 1.**
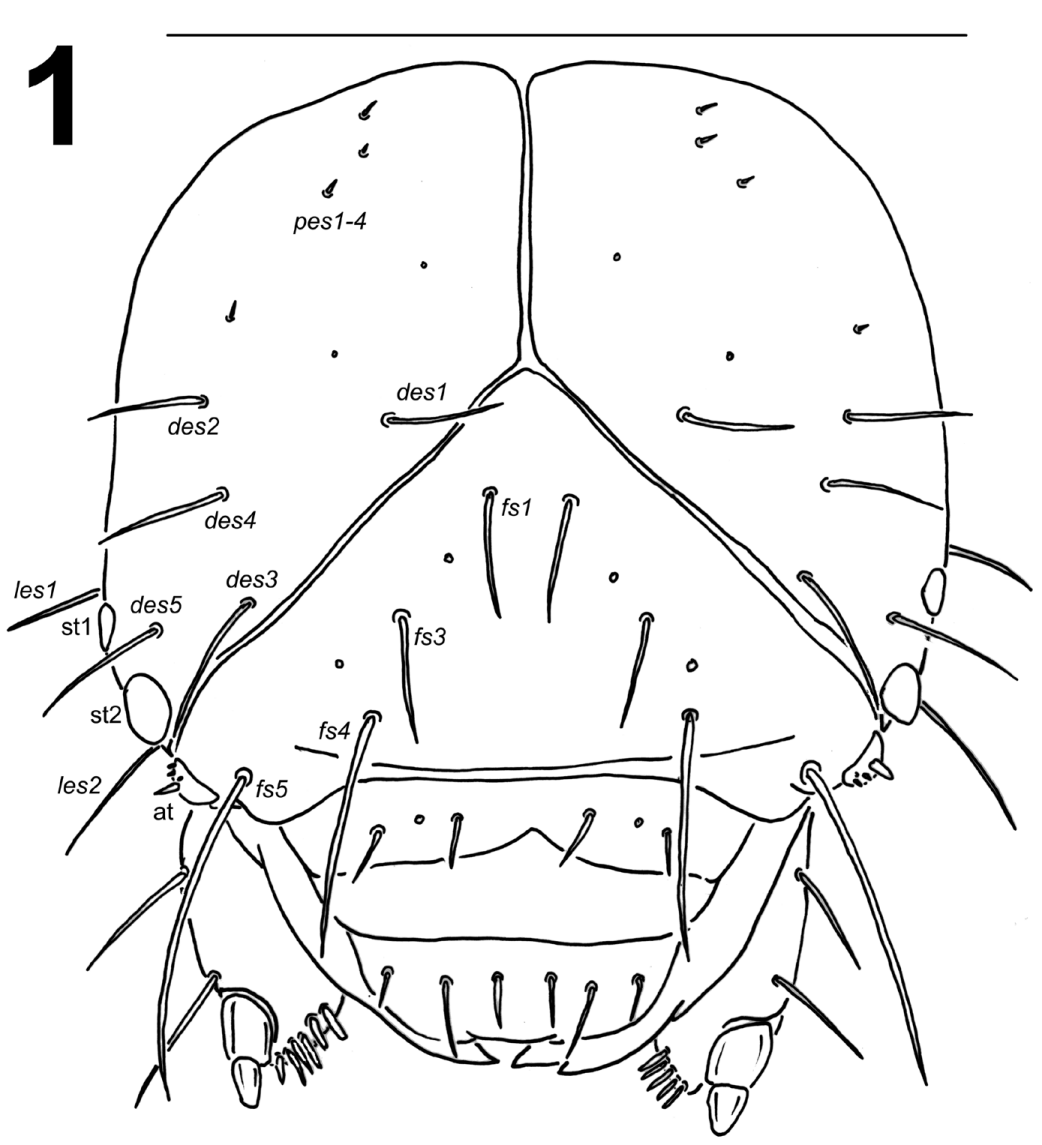
*Metadonus vuillefroyanus* mature larva head, dorsal view. Scale bar: 1 mm.

*Head capsule* (Fig. 1). Head suboval, flattened laterally, endocarinal line absent. Frontal sutures on head distinct, extended to antennae. Two stemmata (st), in the form of a dark pigmented spot with convex cornea, both located on each side anterolaterally, close to each other. *Des1* and *des2* located in upper part of the central part of epicranium, *des1* near to the middle part of epicranium, and *des2* near to side of epicranium, *des3* located anteriorly on epicranium near to frontal suture, *des4* located in the central part of epicranium, *des5* located anterolaterally; all *des* very long, *des3* and *des5* slightly longer than remaining three setae (Fig. 1). *Fs1* and *fs3* placed medially, *fs2* absent, *fs4* located anterolaterally, and *fs5* located laterally, close to the epistoma; all setae long to very long, *fs5* distinctly longer than the remaining four setae (Fig. 1). *Les1–2* as long as *des1*; *ves1–2* short. Epicranial area with four postepicranial setae (*pes1–4*) and two sensilla.

*Antennae* located at the end of the frontal suture on each side, membranous and slightly convex basal article bearing one conical triangular sensorium, relatively long; basal membranous article with three sensilla different in both shape and length (Fig. 5).

**Figures 2–3.**
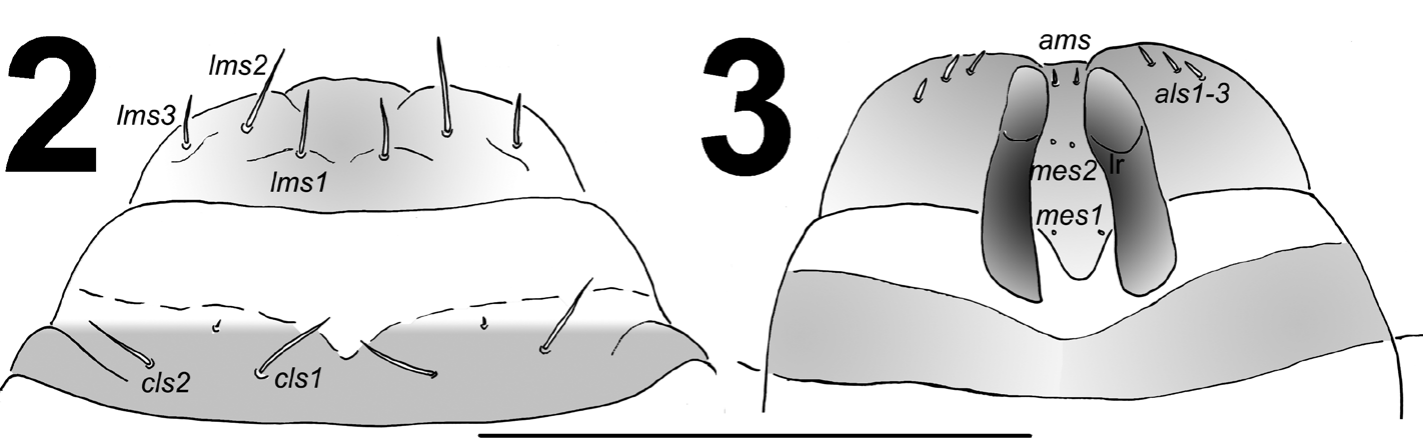
*Metadonus vuillefroyanus* mature larva. **2** Labrum and clypeus **3** Epipharynx. Scale bar: 0.5 mm.

*Clypeus* (Fig. 2) approx. 3 times as wide as long with two relatively long *cls*, almost equal in length, localized posterolaterally, and one sensillum; anterior margin rounded to the inside; median part covered by thorn-shaped cuticular processes.

**Figures 4–5.**
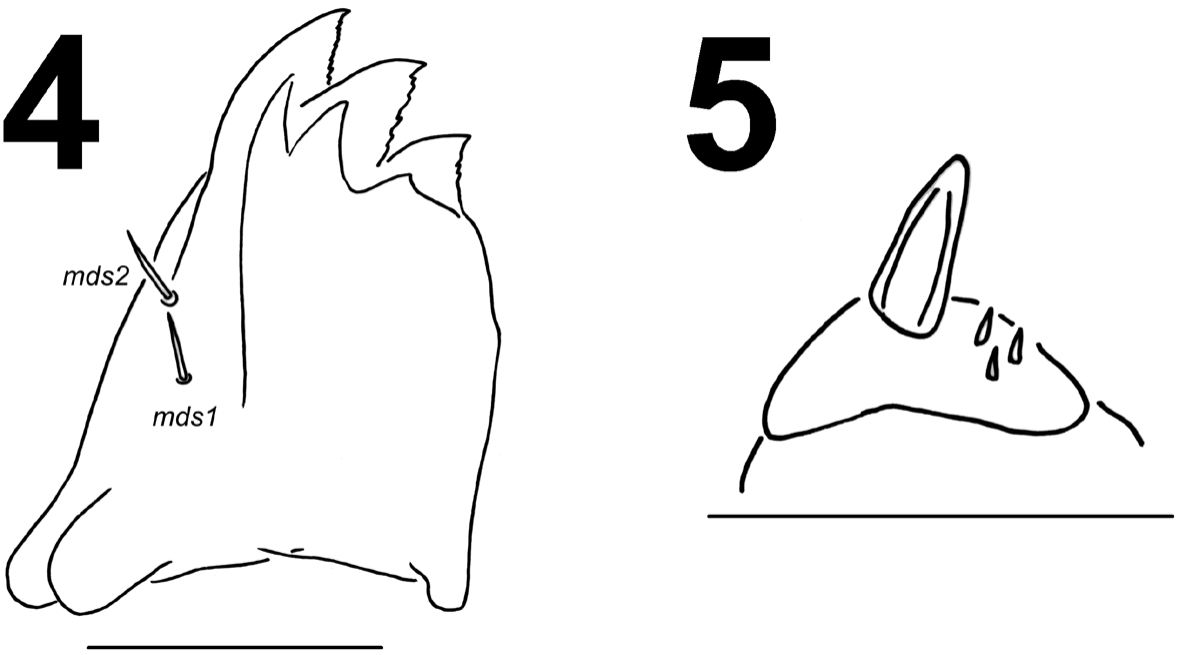
*Metadonus vuillefroyanus* mature larva head. **4** Right mandible **5** Antenna. Scales bars: 0.2 mm (**4**) and 0.1 mm (**5**).

**Figure 6.**
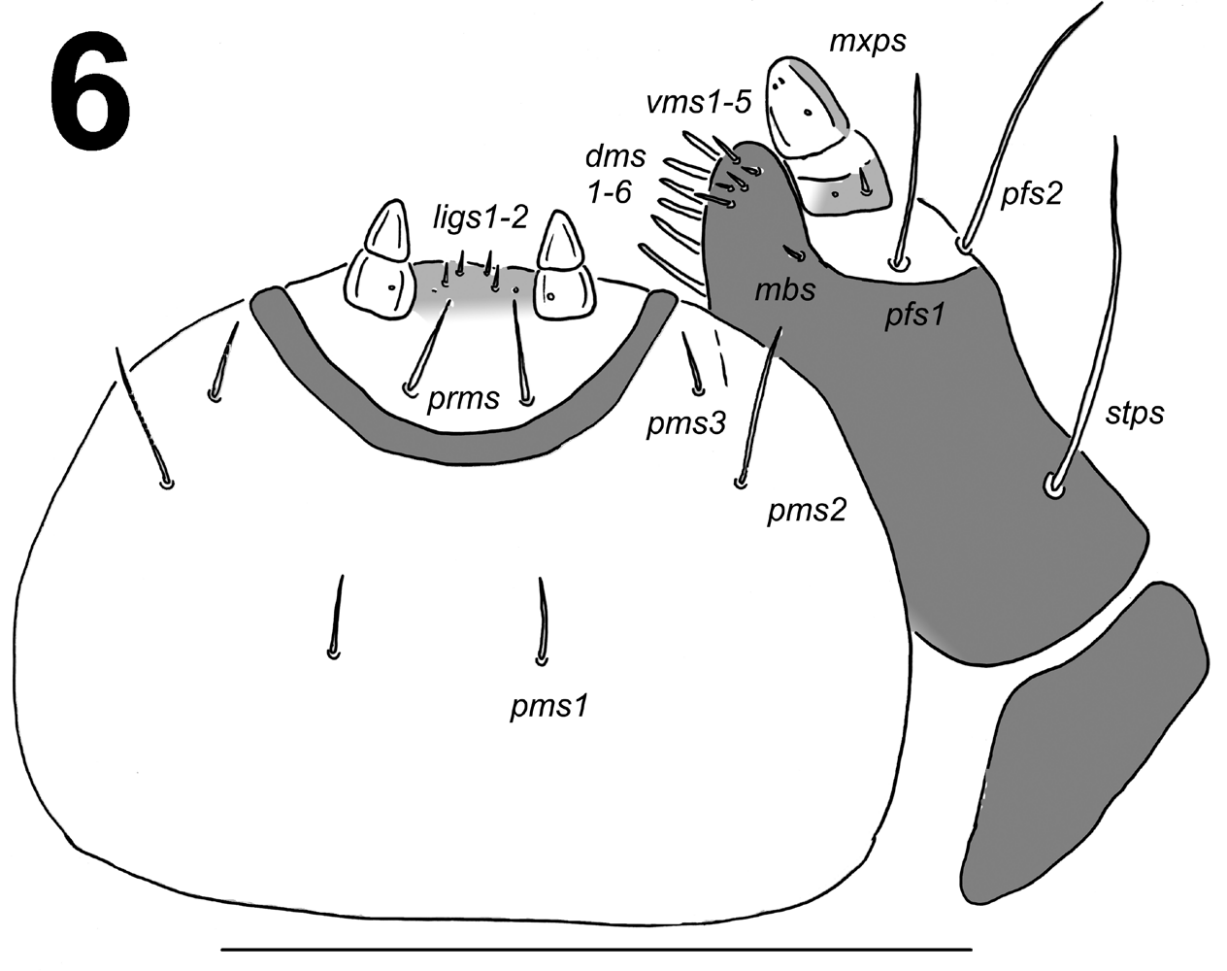
*Metadonus vuillefroyanus* mature larva head, maxillo-labial complex, ventral view. Scale bar: 0.5 mm.

*Mouth parts*. Labrum (Fig. 2) approximately 3.2 times as wide as long, with three pairs of piliform *lms*, of different lengths; *lms3* distinctly shorter than longer *lms1* and *lms2*; *lms1* placed close to the margin with clypeus, *lms2* located anteromedially and *lms3* located anterolaterally; anterior margin double sinuate. Epipharynx (Fig. 3) with three very short, piliform *als*, almost equal in length; one very short piliform *ams*; *mes* not distinct (but apparently there are two setal bases that may correspond to two small setae, not drawn); labral rods (lr) slightly elongated, sub-oval, apical part more sclerotised. Mandibles (Fig. 4) distinctly broad, trifid, tooth of unequal height; slightly truncate; both *mds* relatively long, piliform, located in distinct holes. Maxilla (Fig. 6) stipes with one *stps*, two *pfs* and one *mbs*, *stps* and *pfs1–2* very long, *pfs1* distinctly shorter than *pfs2*, *mbs* very short; mala with six bacilliform *dms*; five very short to minute *vms*, almost equal in length; *vms* distinctly shorter than *dms*. Maxillary palpi with two palpomeres; basal palpomere with one very short *mxps* and two sensilla; length ratio of basal and distal palpomeres: 1:0.8; distal palpomere with one sensillum and a group of conical, cuticular apical processes. Praelabium (Fig. 6) oval-shaped and distinctly elongated, with one relatively long *prms*; ligula with sinuate margin and two piliform very short to minute *ligs*, unequal in length; premental sclerite well visible. Labial palpi with two palpomeres; length ratio of basal and distal palpomeres: 1:0.9; distal palpomere with one sensillum and short, cuticular apical processes; basal palpomere with one dorsal sensillum. Postlabium (Fig. 6) with three *pms*, *pms1* located anteriorly, remaining two pairs laterally; *pms1* and *pms3* relatively long, *pms2* distinctly longer than others; surface of postlabium partly covered by cuticular processes.

*Thorax*. Prothorax distinctly smaller than meso- and metathorax. Spiracle bicameral, placed between the pro- and mesothorax (see Material and methods). Prothorax (Fig. 8) with 11 short to minute *prns* unequal in length, only 3 of them on small weakly pigmented dorsal sclerite, this sclerite subdivided in two triangular plates medially; two short *ps* and one short *eus*. Mesothorax (Fig. 8) with one short and one minute *prs*; four short *pds*; two short *as*; two very short *ss*; one short *eps*; one short and one minute *ps*; and one short and one minute *eus*. Chaetotaxy of metathorax (Fig. 8) almost identical to that of mesothorax. Each pedal area of thoracic segments well separated, with five short to minute *pda*, three of them on pigmented pedal area, unequal in length.

**Figure 7.**
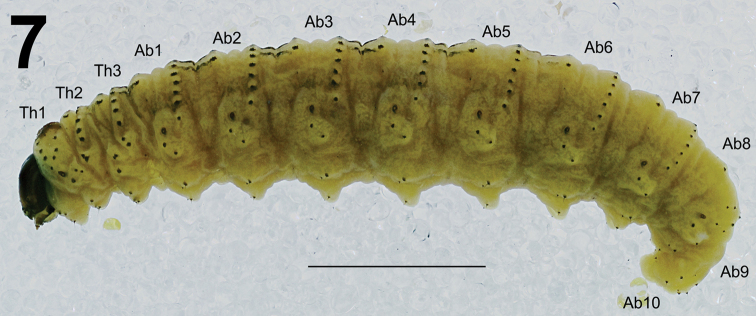
*Metadonus vuillefroyanus* mature larva habitus, lateral view. Scale bar: 3 mm.

**Figures 8–10.**
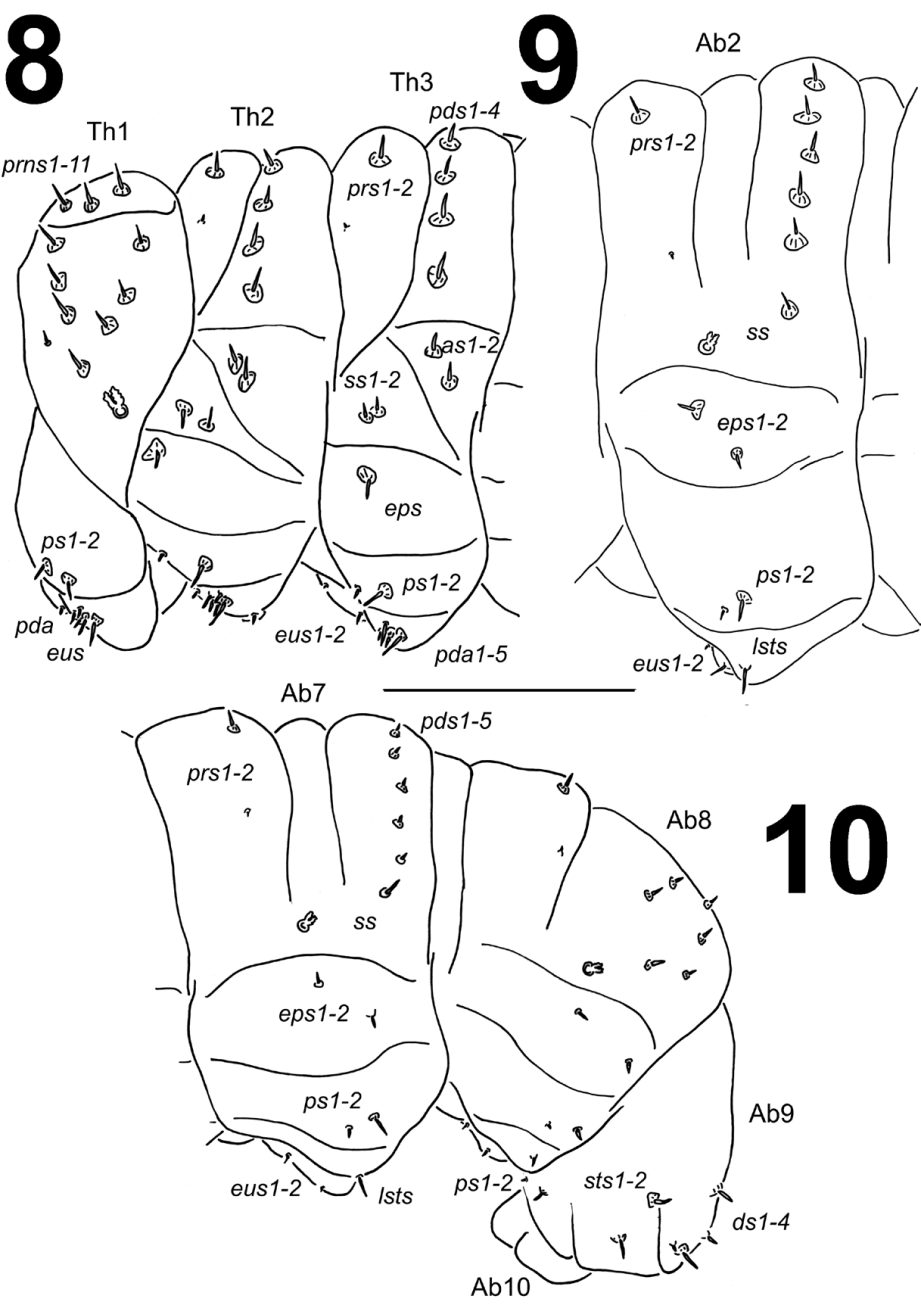
*Metadonus vuillefroyanus* mature larva habitus. **8** Lateral view of thoracic segments **9** Lateral view of abdominal segment II. **10** Lateral view of abdominal segments VII–X. Scale bar: 1 mm.

*Abdomen*. Abdominal segments I–VI of almost equal length, next abdominal segments decreasing gradually to the terminal parts of the body. Abdominal segment X reduced to four anal lobes of unequal size, the dorsal being distinctly the largest, the lateral pair equal in size, and the ventral lobe very small. Anus located terminally; ambulatory ampullae bilobate to circular. Spiracles bicameral, the eight abdominal spiracles located laterally, close to the anterior margin of abdominal segments I–VIII. Abdominal segments I–VII (Figs 9–10) with one short and one minute *prs*; five short *pds*; one short *ss*; two short to very short *eps* of almost equal length; one short and one minute *ps*; one short *lsts*; one short and one minute *eus*. Abdominal segment VIII (Fig. 10) with one very short and one minute *prs*; five very short *pds*, *pds2* and *pds4* not in line; one very short *ss*; two very short *eps* of almost equal length; one very short and one minute *ps*; one very short *lsts*; and one very short and one minute *eus*. Abdominal segment IX (Fig. 10) with four *ds* (two *ds* very short, two *ds* minute); two very short *ps*; and one very short and one minute *sts*. Abdominal segment X (Fig. 10) without setae.

#### Description of pupa.

*Measurements* (in mm). Body length: 7.0–8.0 (♂ 8.0; ♀ 7.0); at the widest region: 4.5–5.0. The widest place in the body is commonly between the apex of the meso- or metafemora. Unfortunately, both pupae were damaged and the measurements are not precise.

*Colouration*. Body yellowish with greenish abdomen (Figs 17, 19).

**Figures 11–13.**
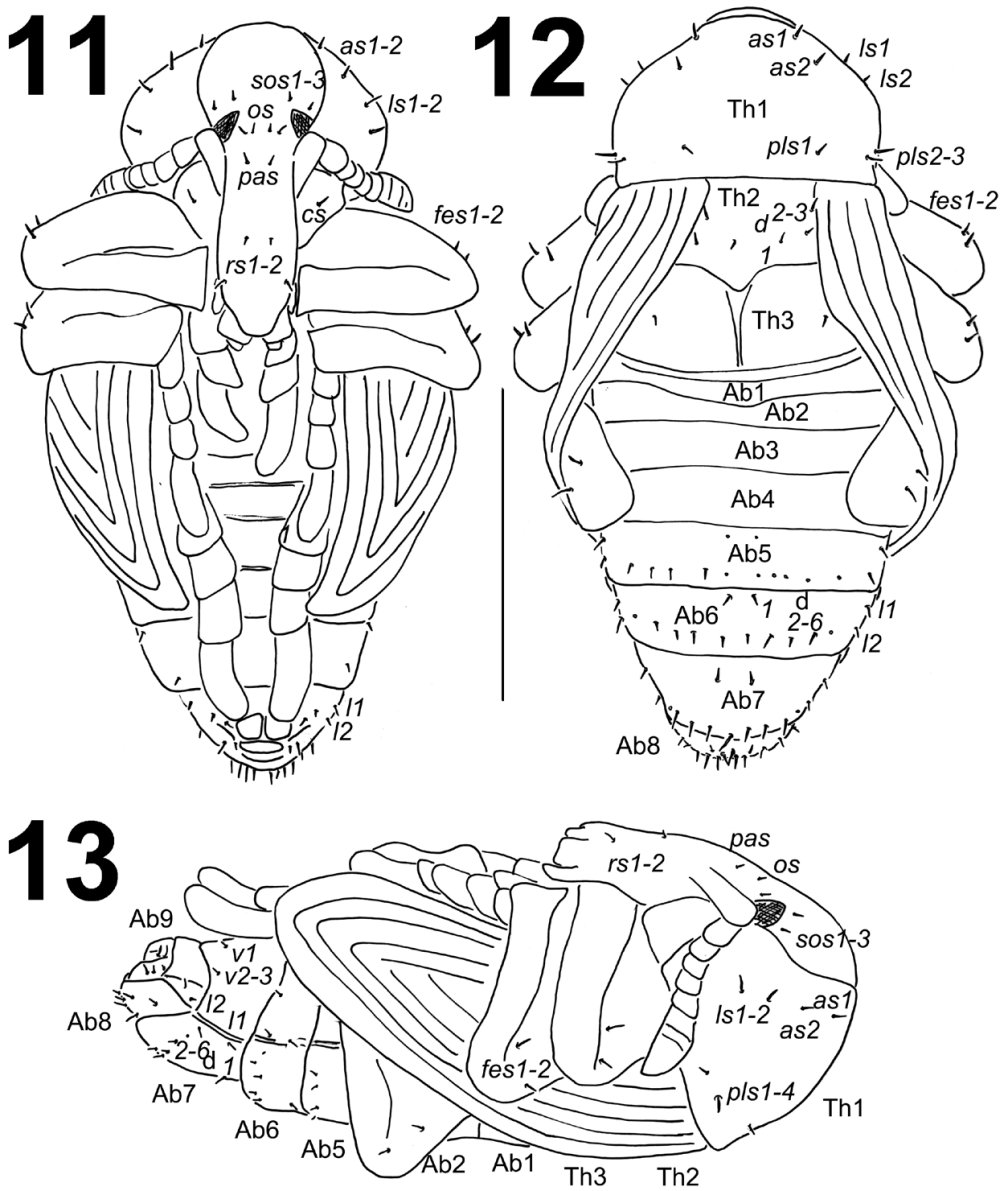
*Metadonus vuillefroyanus* pupa habitus. **11** Ventral view **12** Dorsal view **13** Lateral view. Scale bar: 3 mm.

*Morphology* (Figs 11–13). Body stocky, cuticle smooth. Rostrum relatively long, approximately 2.5 times as long as wide, extended to metacoxae. Antennae relatively long and stout. Pronotum from 1.7 to 1.8 times as wide as long. Mesonotum and metanotum of almost equal length. Abdominal segments I–IV of almost equal length; abdominal segment V semi-circular, next abdominal segments diminish gradually to the end of the body. Abdominal segments VI–IX distinctly smaller than other abdominal segments. Gonotheca (abdominal segment IX) in females (one specimen) divided. Sexual dimorphism in weevil pupae is visible mainly in the length of rostrum and in the structure of abdominal segment IX: gonotheca of ♂ undivided and divided in ♀.

*Chaetotaxy* (Figs 11–13). Setae short to very short, unequal in length, light yellow, orange up to black. Setae well visible. Unfortunately, both pupae were damaged and it was not possible to observe chaetotaxy on some parts of body. Head capsule includes only three *sos*, one *os* and one *pas*. Rostrum with two *rs*. Setae on head capsule straight, as short as the remaining setae on thoracic and abdominal segments. Pronotum with two *as*, two *ls* and three *pls*, *ds* not observed. Dorsal parts of mesothorax with one seta located posteromedially, one seta posterolaterally and one seta located along its anterior margin. Dorsal parts of metathorax with one seta located along its anterior margin. Coxa with one very short seta (*cs*). Each apex of femora with groups of two *fes*. Abdominal segments I–V damaged, and it was not possible to observe setae. Dorsal parts of abdominal segments VI–VIII each with one seta located posteriorly (*d1*) and five pairs (*d2–6*) located along their anterior margins; setae short, hair-like. Abdominal segments V–VIII with groups of two lateral setae and one (or two) ventral setae. Abdominal segment IX with two ventral microsetae and two short, thin setae. Pseudocerci absent.

**Figures 14–17.**
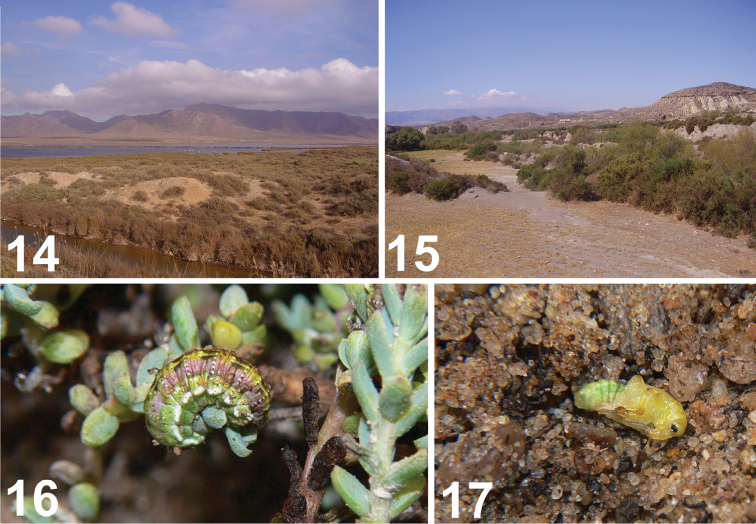
Habitat and immature stages of *Metadonus vuillefroyanus*. **14** Locality Cabo de Gata **15** Locality Tabernas **16** Mature larva on host plant, **17** Pupa on ground. Photos: Bogusch P (**13**, **14**), Pelikán J (**16**, **17**).

#### Biological notes.

*Habitats*. *Metadonus vuillefroyanus* occurs only at saline sites in the presence of its host plant. This species has been observed both at saline sites near the sea (locality Cabo de Gata less than 1 km from the seashore, Fig. 14) and at inland saline sites (Tabernas is approximately 50 km inland from the sea, Fig. 15). The population density of the host plant was not high at these localities; at Cabo de Gata it was much less numerous than the dominant *Salicornia maritima*, and in Tabernas only several plants were observed. The ruderal sites containing the host plant have also been controlled but without any observations of *Metadonus vuillefroyanus*.

*Adult behaviour*. Adults are only occasionally present on host plants during the day; most of them are hidden under the plant and are active at night.

*Host plant*. Both adults and larvae were observed feeding exclusively on *Suaeda vera* (Fig. 16). The larvae were found directly on the plants during the day, usually near the growth terminals. Adults and larvae feed on the leaves of the host plant. Larvae, with their cryptic pattern, resemble the colouration of the leaves (Fig. 16). The larvae were found on larger (more than 30 cm high) bunches of the host plant and they usually settled on those with thick, old, dry branches. The adults probably do not migrate long distances and remain under the plant or group of plants at their site.

**Figures 18–21.**
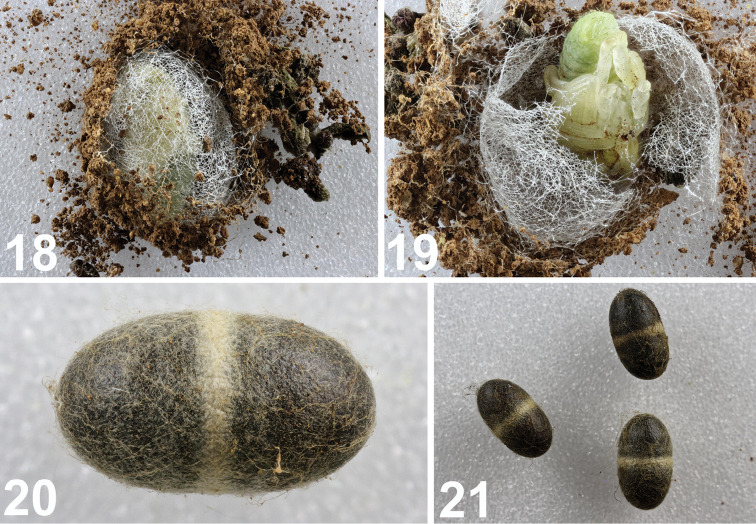
Cocoon, pupa and parasitoids of *Metadonus vuillefroyanus*. **18** Cocoon with two layers **19** Pupa in cocoon **20** Detail of parasitoid’s cocoon **21** Cocoons of parasitoids. Photos: Bogusch P (**18–21**).

*Life cycle*. At the beginning of April, 15 mature larvae and five younger larval instars were swept from host plants. At that time, the host plants had quite young fresh leaves, so the development of these individuals probably began a few days or weeks earlier in the spring so larvae would emerge on the young leaves of the host plant. We suppose the whole larval development to be short-lasting, approximately three weeks at most. The larvae were kept in small plastic containers (height 12 cm, width 4 cm) with small holes for air circulation and exchange. Four to five larvae were kept in each container and no aggressive behaviour was observed, as is known in some Hyperini, e.g., *Brachypera vidua* (Gené, 1837) (Skuhrovec et al. 2015b). The larvae started pupation early, usually within 2–3 days. The bottom of each container included a 5 cm high level of substrate (collected directly at the locality under the host plants). The majority of the larvae spun their cocoons on the bottom of the container; therefore, they probably generally pupate less than 5 cm into the ground in natural conditions. However, several larvae made the cocoons just below the substrate level, so there seem to exist differences in the pupation preferences (Fig. 18). These cocoons were usually under dead chunks of *Suaeda* provided for feeding. None of the larvae pupated on or above the ground. The process of creating the cocoon took approximately 1–3 days. Subsequently, larva stayed in the cocoon until pupation, which occurred after 2–4 more days. Most of the larvae moulted into pupae within 5–7 days after entering the substrate. Adults began to emerge 12–18 days later. Altogether, seven adults emerged (five males and two females) by biting a hole in the cocoon. The new adults stayed on the ground surface or remained between a few millimetres and 3 cm above the ground. They were light and smooth at first, but soon acquired the general appearance of normal mature insects, even though it took 3–5 days for them to fully sclerotize. The adults fed on thawed host plant material brought from Spain, but the plants were damaged and did not supply sufficient food. Due to the absence of these host plants in the Czech Republic, we did not try to breed the insects and obtain eggs for a new generation.

The pupae have a green colouration that changes to a brownish colour approximately five days after the start of pupation (Fig. 19). The colouration changes over the entire body; only the eyes are slightly darker. The adults are dark brown (in contrast to the sandy light brown of newly emerged imagines) by 10 days after pupation and are ready to emerge. The emerging adults usually stay in the cocoon for 1–2 days until they are partly sclerotized and then come out and feed on the host plant. The cocoon has two silk layers. The inner layer protects the pupa and is made only of silk. The outer layer includes ground particles.

*Biotic interactions*. Several of the larvae did not pupate, but larvae of parasitoids emerged and formed typical dark-brown puparia with a whitish band (Figs 20–21).

## Discussion

### Comparison with immature stages of other Hyperini

The larvae of 43 hyperine taxa have already been described (Anderson 1948; Baccetti 1957, 1958, 1959; Zaslavskij 1959, 1965, 1967; Scherf 1964; Lee and Morimoto 1988; May 1993; Nazarenko 2000a, b; Marvaldi 2003; Skuhrovec 2005a, 2006a, 2007; Vanin et al. 2012). The detailed description of the pupa is similar for nine hyperine taxa (Baccetti 1957, 1958, 1959; Scherf 1964; Gosik 2008; Vanin et al. 2012).

The comparison of the larvae and pupae of *Metadonus vuillefroyanus* with those described by Scherf (1964) was somewhat difficult due to the use of different terminology for morphology and chaetotaxy and/or the absence of good-quality drawings. The larvae were compared with the majority of species described and/or drawn by Anderson (1948), Zaslavskij (1959, 1965, 1967), Scherf (1964), and Nazarenko (2000a, b) which are of high or good-enough quality and very useful; however, the described characteristics are useful only for differential diagnosis. Detailed descriptions of these hyperine taxa are missing.

Only one brief description of *Metadonus distinguendus* were done by Zaslavskij (1959), being the only previously known in the genus *Metadonus*. Unfortunately, the description includes only drawings of the dorsal view of the prothorax, mesothorax and abdominal segment II. Despite these challenges, we were able to compare the morphology of these two taxa. *Metadonus vuillefroyanus* has one more minute *prn*, one more minute *prs* on mesothorax (see Fig. 8 for both) and two more *prs* (one normal size and one minute, see Fig. 9) on abdominal segment II than larvae of *Metadonus distinguendus*. Both species have a unique characteristic in that all setae on the larval body are thorn-like and located on distinct black protuberances. This feature is also known in hyperines in all four species of the subgenus *Eririnomorphus* Capiomont, 1868, for which larvae are known (Anderson 1948; Skuhrovec 2006a, b) as well as in *Hypera arator* (Linnaeus, 1758) of the subgenus *Kippenbergia* Alonso-Zarazaga, 2005 (Skuhrovec 2005a). Species of the subgenus *Eririnomorphus* have more points of similarity with species of the genus *Metadonus* than just these specific setae with protuberances (Skuhrovec, unpubl. data). The adults of these two groups have very similar body-scale shapes (see Skuhrovec 2008, 2009) and also share particularly harsh or extreme habitats (Skuhrovec 2009, 2012). The species of subgenus *Eririnomorphus* are more similar in these characteristics to *Metadonus* than to any other species of the genus *Hypera* (Skuhrovec, unpubl. data). This is suggestive of a close phylogenetic relationship between these taxa, a hypothesis that remains to be tested via phylogenetic analyses of the hyperines.

Various morphological characteristics of larvae of the tribe Hyperini were published by Lee and Morimoto (1988), May (1993), and Marvaldi (2003), including epipharynx and maxilla with simple setae, the third dorsal seta (*des3*) on the epicranium, the fifth frontal seta (*fs5*) longer than the fourth one (*fs4*), labial palpus one-segmented, mandible with sharp teeth, labral rods indistinct, postoccipital condyles present, pedal areas swollen to form prolegs or large lobes, head maculate and pigmented body. Vanin et al. (2012) published a detailed description of the immature stages of *Phelypera schuppeli* (Boheman, 1834) and found that the larvae have 2-segmented labial palpi unlike “typical” hyperines, but identical to *Metadonus vuillefroyanus*. A comparative summary of all recent data was provided by Oberprieler et al. (2014). Later, Nazarenko (2014) described and discussed the epipharyngeal morphology of seven hyperines. His final main epipharyngeal composition, with three pairs of *als*, one (or two) pairs of *ams* and two pairs of *mes*, completely fit the morphology of *Metadonus vuillefroyanus* (Fig. 3). The precise count of some of the setae on the epipharynx (especially *ams* and *mes*) in weevils is not completely resolved. According to Marvaldi (1998a, 1999), the standard for epipharynx setae in weevils is two *ams* and three *mes*, but when the positions of the distal *mes* are very close to the anterior margin they appear as *ams* (see different solutions within the same groups, e.g., Tychiini: Skuhrovec et al. 2014 vs. 2015b).

Knowledge of the immature stages and life histories of species can help to protect endangered species more effectively. The detailed description of larvae and pupae and their comparisons with known descriptions as reported here demonstrates that it is possible to identify this species in its immature stages, as has been accomplished for other groups (i.e., Entiminae: Gosik and Sprick (2012a, b, 2013); Gosik et al. (in press); Curculioninae, Tychiini: Skuhrovec et al. (2014, 2015b); Lixinae: Gosik and Skuhrovec 2011; Gosik and Wanat 2014; Stejskal et al. 2014; Skuhrovec and Volovnik 2015; Trnka et al. 2015). This process is particularly valuable for rare and endangered species because finding larvae is typically much simpler than finding adults. Additional detailed descriptions for hyperines, precise keys, detailed generic studies and comparisons of all groups could help in future to undertake the phylogenetic analysis of this group. It could also be very useful in different entomological fields, such as agriculture, biological control, and protection of endangered species.

### Biological singularity

Hyperines are mainly known for two typical but biologically unusual features: ectophytic larvae with cryptic colouration and the ability to spin mesh cocoons. Both these features have been confirmed in *Metadonus vuillefroyanus* with specific details. Velázquez de Castro et al. (2000) presented the first biological data for *Metadonus* species, particularly for *Metadonus vuillefroyanus*, stating that this weevil occurred in salty wetlands, and that its host plant was *Suaeda vera*, which is a common plant in salty wetlands on Mediterranean seacoasts. All these data correspond with our observations.

Skuhrovec (2012) described J. Krátký’s and J. Pelikán’s observations of this weevil in salt wetlands in southeastern Spain. They described the colouration of larvae as similar to other Hyperini larvae – greenish with white stripes; however, these larvae have a thick yellow stripe and parallel pink to violet stripes on their dorsal parts (see Description and Fig. 16). Our observations about larval colouration are similar to previous descriptions, but the observations differ in the intensity and extent of purple stripes on the dorsum of the larvae. Some larvae were more green than purple, but some larvae were completely purple on the dorsal and lateral parts of their bodies. The colouration of the purple stripes varied from dark red to dark violet, almost black in some cases. The width of the dorsal yellowish stripes was also quite variable. A similar variation was observed in the plants, the shrubs of *Suaeda vera*: some were completely green, most were green and reddish, and some had completely reddish or violet-coloured leaves. We think that the variable colouration of the larvae corresponds with the colouration of the leaves of the host plant on which they fed, but we did not study this topic in detail (for example, whether the larvae are redder on reddish plants than on green ones). Cryptic colouration is one of the most common defensive strategies among insects and their larvae (Alcock 2009). Hyperine larvae are among the few in weevils that feed externally on the surface of their host plants, which may explain why they have evolved this protective colouration. The cryptic coloration, among other features like reduced body setae, and ambulatory ampullae, is also found in other weevil groups with ectophytic larval feeding, like *Gonipterus* and *Listroderes* (May 1993, 1994; Marvaldi 1998b).

Skuhrovec (2012) claimed that his colleagues observed that larvae of this species do not create cocoons; instead, they dig into the soil. Our observations are unambiguously different and we can rectify the previous mistake. According to the observations of the second author of this paper, the cocoon has two layers (Fig. 18). The inner layer protects the pupa and is made only of silk spun from Malpighian tubules. The outer layer is similar to the inner layer but the surface is covered with soil particles. This is different from cocoons of other similar and related European genera and species (Skuhrovec, unpublished data), which usually pupate on or above the ground and do not use substrate particles to build cocoons. The function of the outer part of the cocoon is probably a defensive tactic against parasitoids as camouflage or more likely, against very dry and/or very wet site weather conditions. The outer layer was also observed earlier by Krátký and Pelikán (Skuhrovec 2012), but the inner layer was overlooked. Some of the larvae collected for this study were parasitized by still-unidentified braconids (they formed typical dark brown puparia with whitish bands, see Fig. 20); this observation lends more support to the hypothesis that the outer cocoon layer’s function is for protection against unstable weather conditions.

## Supplementary Material

XML Treatment for Metadonus vuillefroyanus
